# Perfluorooctane Sulfonate (PFOS) Disrupts Mitochondrial Activity and Cell Adhesion in Liver Cells

**DOI:** 10.3390/jox16020065

**Published:** 2026-04-13

**Authors:** Phuong D. Tran, Kyoungtae Kim

**Affiliations:** Department of Biology, Missouri State University, 901 S National Ave, Springfield, MO 65897, USA; pdt8s@login.missouristate.edu

**Keywords:** PFOS, HepG2, THLE-2, mitochondrial damage, CAMs, cell adhesion, ATP, OXPHOS, apoptosis

## Abstract

Perfluorooctane sulfonate (PFOS) is a persistent environmental pollutant associated with potential hepatoxic effects and other health risks. Despite its widespread distribution, the mechanisms underlying its toxicities remain to be fully understood. To investigate PFOS toxicology, our study utilized HepG2 and THLE-2 human hepatic cell models to replicate conditions reflecting PFOS accumulation in the liver. Cell viability, cell stress, and cell death assays were conducted to assess the toxicological influence of the chemical on both cell lines. Total RNA extraction was performed, followed by cDNA sequencing, and rt-qPCR. The XTT viability assay revealed a dose-dependent decrease in the number of viable cells when incubated with increasing concentrations of PFOS. The inhibitory concentration (${IC}_{50}$) values were approximately 100 micromolar, which led to morphological changes, elevated reactive oxygen species (ROS), and induced early apoptosis in liver cells after 6 h. Based on the transcriptomic analysis for HepG2 cells, mitochondrial genes involved in oxidative phosphorylation were downregulated, including COX, ND, and the ATP synthase family. Additionally, significant alterations of transcripts implicated in cell adhesion molecules (CAMs) were observed. In conclusion, PFOS inhibited cell growth, induced oxidative stress, and elevated apoptotic levels via transcriptomic alteration, including gene transcripts required for mitochondrial activity and cell adhesion.

## 1. Introduction

Per- and polyfluoroalkyl substances (PFASs) are a group of man-made chemicals that [1,2] are nonreactive, amphiphilic, oil-repelling, and resistant under extreme conditions [3]. In the industry, PFASs were produced by electrochemical fluorination (ECF) where hydrogen atoms were replaced with fluorine or telomerization [1,4]. Due to exceptionally strong carbon-fluorine covalent bonds, about 100 kcal/mol [1], PFASs are highly stable [5]. Therefore, they are used in the production of numerous products such as surfactants, textile stain, soil repellents, insecticides, non-stick cookware, food packaging, aqueous film-forming foam (AFFF), and cosmetics [3,5]. Despite their unique profiles, PFASs have raised significant concerns as emerging pollutants, with increasing documentation of their negative impacts on both abiotic and biotic factors [2,6,7].

Of 26,943 PFASs in the regulatory collection [8], perfluorooctane sulfonate (PFOS) has been the most widely detected [9,10], despite its large phase-out in the early 2000s [11,12]. PFOS has been found in various environmental matrices and has been reported to enter into the food web and water supplies [10,13,14]. In human matrix, PFOS was detected in the serum of residents and workers in North America, Asia, and Europe within the range from 5.89 ± 3.73 to 30.26 ± 53.11 ng/mL [15]. Additionally, PFOS accumulates in the placenta [16,17], associating with adverse health issues in pregnant mothers, and affecting fetal development [18]. Animal studies have demonstrated PFOS toxicities on the liver, immune, nervous, developmental and thyroid systems [19,20,21,22,23]. However, the liver is recognized as the primary site of PFOS toxicological study [24,25,26,27,28]. Multiple epidemiological studies, such as a study from the C8 Health Project in American adults (*n* = 46,452), suggested a positive relationship between PFOS exposure and the increasing levels of alanine aminotransferase (ALT) in serum, a key biomarker of hepatocellular damage [29,30,31,32]. Another study including the epidemiological research of two different female populations in China (*n* = 883) and the U.S. (*n* = 5845) showed a positive association between PFOS and liver injury, evidenced by an elevated FIB-4 index which estimates the degree of liver fibrosis [33]. In 2022, a nested case–control study (*n* = 100) by Goodrich et al. revealed that high PFOS levels (>55 μg/L) were associated with 4.5-fold higher risk of hepatocellular carcinoma (HCC). Metabolomic analysis suggested that PFOS exposure was associated with alterations in glucose, amino acid, and bile acid metabolism [34]. Additionally, animal studies pointed out that PFOS can induce hepatocellular enlargement, vacuolation, necrosis, hepatic steatosis, and hepatic fat accumulation [19,20,25,35,36,37]. In vitro studies have shown significant elevation of reactive oxygen species (ROS), induced oxidative DNA damage [38,39], disruption in PPARα [40] and altered lipid transport and β-oxidation in human hepatocytes HepG2 [41]. Despite its potential toxic effects, the mechanisms underlying PFOS toxicities on the liver are yet to be fully elucidated.

In the current study, we used liver cell models to study PFOS toxicity and xenobiotic metabolism. HepG2 is a popular human-derived hepatoma cell line that has been used to study a wide range of applications in scientific research, from oncogenesis to the hepatotoxicity [42]. In addition to HepG2 cells, we also included THLE-2 as a non-tumorigenic, more physiologically relevant hepatic model. THLE-2 cells are derived from normal adult human liver epithelium and immortalized via the introduction of SV40 large T antigen, allowing extended proliferation while retaining key characteristics of healthy hepatocytes [43]. Therefore, by incorporating THLE-2 alongside HepG2 cells, the current study aims to distinguish PFOS-induced responses in different hepatic models and to improve the translational relevance of in vitro liver toxicity assessments.

In brief, cell viability, intracellular reactive oxygen species (ROS), and apoptosis assays were used to assess PFOS toxicological impacts. Transcriptomic analysis, followed by RT-qPCR confirmation and ATP assay, suggested that PFOS could disrupt mitochondrial activity and cell adhesion in human liver cells.

## 2. Materials and Methods

### 2.1. Materials

The HepG2 cell line (ATCC HB8065) and THLE-2 cell line (ATCC CRL-2706 TM) were obtained from American Type Culture Collection (Manassas, VA, USA). Dimethyl sulfoxide (DMSO) (Cat No. D1391), and 2,7-dicholorofluorescein-diacetate (DCFH-DA) (Cat No. D399) were purchased from Fisher Scientific (Waltham, MA, USA). HyClone Dulbecco’s Phosphate Buffered Saline (DPBS) (Cat No. SH30028.03), and HyClone RPMI-1640 medium (Cat No. SH30027.02) for HepG2 cells were obtained from Cytiva (Marlborough, MA, USA). XTT reagent (Cat No. 30007) was from Biotium (Fremont, CA, USA), and heptadecafluorooctanesulfonic acid solution (PFOS) (Product No. 77283) was from Sigma-Aldrich (St. Louis, MO, USA). Heat-inactivated fetal bovine serum (FBS) (Product No. 35-016-CV), penicillin–streptomycin solution (pen–strep) (Product No. 30-002-CI), and 0.25% trypsin-EDTA solution (Product No. 25-053-CI) were obtained from Corning (Manassas, VA, USA). Obtained from Lonza Bioscience (Basel, Switzerland) was the culture medium for THLE-2 cells that contained BEBM Bronchial Epithelial Cell Growth Basal Medium (CC-3171) and BEGM Bronchial Epithelial Cell Growth Medium SingleQuots Supplements and Growth Factors (CC-4175).

### 2.2. Cell Culture

HepG2 and THLE-2 cells were cultured in 25 ${cm}^{2}$ tissue culture flasks and incubated at 37 °C and 5% ${CO}_{2}$. HepG2 flask was supplemented with 10% FBS and 1% antibiotics in RPMI media, while THLE-2 flask was supplemented with 10% FBS and 1% antibiotics in BEGM media. The media for each flask were replaced twice weekly, and cultures were split when they reached 80% confluence.

### 2.3. Cell Viability

We determined cell viability after exposure to varying concentrations of PFOS using XTT assay. HepG2 and THLE-2 cells were seeded into 96-well plates at a density of 10,000 cells/well and incubated overnight. Cells were then exposed to PFOS at the concentrations of 30, 60, 90 and 120 μM for 24 h. After exposure to the chemical, the supernatant was discarded, cells were washed once with 1× PBS. Afterwards, 100 μL XTT reagent was added to each well. The plates were incubated in the dark for 3 h at 37 °C. Afterwards, we measured the absorbance at 540 nm using a spectrophotometer (BioTek, Winnooski, VT, USA). A positive control of 20% DMSO was included. Each treatment was tested in hexaplicates.

### 2.4. Morphological Studies and Cell Attachment Analysis

We used an Olympus IX81 Motorized Inverted Research Microscope to study morphological changes in HepG2 upon PFOS exposure. In brief, HepG2 cells were seeded into a μ-slide 18-well plate (Cat No. 81816, ibidi GmbH, Grafelfing, Germany) at a density of 10,000 cells per well and incubated overnight. Cells were then exposed to PFOS at 112 μM for 6, 12, or 24 h. After exposure, we discarded the medium, added 100 μL clear DMEM to each well, and observed the cell morphology under the microscope. Differential interference contrast (DIC) images were captured at 200× magnification. The number of intact cells was determined using the hemocytometer and normalized to percentage values. Each treatment was tested in triplicate.

### 2.5. Intracellular ROS Formation

We measured intracellular ROS formation in HepG2 cells using DCFH-DA (2′,7′-dichlorofluorescein diacetate) (Cat No. D399, Thermo Fisher Scientific, Waltham, MA, USA). In brief, HepG2 cells were seeded into a μ−slide 18-well plate at a density of 10,000 cells per well and cultured overnight. Cells were then exposed to PFOS at 112 μM for 6 h, washed once with 1× PBS, and incubated with DCFH-DA for 40 min in the dark at 37 °C. We visualized the cells using a fluorescence microscope and used Image J to quantify the fluorescent intensity. A positive control of 20% DMSO was included. Each treatment was tested in triplicate.

### 2.6. Apoptosis Assays

We measured apoptotic level in HepG2 cells using Annexin V/Propidium Iodine (PI) assay (BD Biosciences, San Jose, CA, USA). Cells were seeded at a density of 70,000 cells/well in a 12-well plate and incubated overnight. Cells were then treated with 112 μM PFOS for 6 h. After that, HepG2 cells were detached using Trypsin without EDTA, collected into Eppendorf centrifuge tubes, and stained with Annexin V/PI for 30 min in the dark at 37 °C. After incubation, 1× Annexin V binding buffer was added to each tube to the final volume of 500 μL. We used an Attune NxT flow cytometer (Thermo Fisher Scientific, Waltham, MA, USA) to collect and analyze the samples. Each treatment was tested in triplicate.

### 2.7. RNA-Sequence Analysis

We performed a total RNA extraction on HepG2 cells. First, the cells were seeded into a 6-well culture plate with a density of 2,000,000 cells/well and incubated overnight. Cells were then exposed to PFOS at 112 μM for 6 h. After the chemical treatment, HepG2 cells were homogenized using TRIzol method to isolate total RNA. RNA quality and integrity were assessed using Agilent 4150 TapeStation Software v5.2 (Agilent Technologies, Santa Clara, CA, USA), with RINe ≥7.0 indicating as a pure sample, and confirmed by agarose gel electrophoresis. Purified RNA was sent to Novogen Lab (Sacramento, CA, USA) for sequencing. After obtaining the data, we proceeded to transcriptomic analysis using CLC Genomics Workbench 24.0.3 (QIAGEN). Differential expressed genes (DEGs) with significance were sorted based on ${log}_{2}fold\;change$ (${log}_{2}FC$) ≥ 2 for upregulated genes and ${log}_{2}FC$ ≤ −2 for downregulated genes, with a false discovery rate (FDR)-adjusted *p*-value ≤ 0.05. Hierarchical clustering was shown using the Heatmap function.

### 2.8. PANTHER and KEGG Pathway Classification

Differentially expressed genes (DEGs) were functionally annotated using PANTHER classification system to categorize genes into biological process pathways. The resulting gene lists were used to generate functional classification diagrams. Selected DEGs associated with biological processes of interest were subsequently imported into KEGG database for pathway mapping and visualization.

### 2.9. RT-qPCR

For RT-qPCR, CYP7A1 and HSPA6 genes were selected for RNA sequence validation. We used the ${SuperScript}^{TM}$ IV First-Strand Synthesis System Kit (Invitrogen, Waltham, MA, USA, CAT NO: 18091050) to synthesize cDNA. Forward and reverse primers were ordered from Integrated DNA Technologies (IDT, Coralville, IA, USA). First, an efficiency test was carried out to ensure primer quality. Subsequently, 800 ng of purified RNA from each sample was used for cDNA synthesis. Newly synthesized cDNA concentrations were then quantified using ssDNA Qubit Assay Kit (CAT NO: Q10212, Invitrogen, Thermo Fisher Scientific, Waltham, MA, USA). We used Applied Biosystems QuantStudio 5 Real-Time PCR System (Thermo Fisher Scientific, Waltham, MA, USA) for cDNA amplification. $C_{T}$ values were obtained from QuantStudio Design & Analysis Software v1.5.2 (Thermo Fisher Scientific, Waltham, MA, USA). Earlier amplification of the selected gene in treated samples relative to controls indicated increased gene expression, while delayed amplification indicated decreased expression. Comparable $C_{T}$ values for the housekeeping gene across treated and control groups verified the validity of the assay. Relative fold change was calculated using the double delta ($2^{-∆∆CT}$) method [44].

### 2.10. ATP Assay

We used a Steady-ATP HTS Viability Assay Kit (PSF006, Biotium, Fremont, CA, USA) to measure ATP levels in HepG2 cells. A standard curve was created before the process. In brief, we made the original stock with 2 mM ATP standard and had the highest concentration of ATP at 500 pmol per 100 μL. A 1/10 serial dilution was carried out five more times. Afterwards, we added 50 μL of each concentration into a 96-well plate, then added 50 μL of ATP Assay Mix. We repeated triplicates for each concentration. The mixture was then collected into Eppendorf tubes, and measured for relative light units (RLUs) using a luminometer (Berthold Technologies, Bad Wildbad, Germany). The average of RLUs was taken, and converted into logarithm base 10. A graph was created using log (RLU/s) on y-axis, and ATP concentration gradients (0.005, 0.05, 0.5, 5, 50, and 500 pmol) on x-axis. We then added the equation correlating to all the datapoints, and achieved a $R^{2}$ = 0.9716. The equation was used to calculate for the actual experiment.

On the day proceeding the ATP measurement assay, cells were seeded into a 96-well plate with a density of 10,000 cells/well and incubated overnight. Subsequently, cells were exposed to PFOS at 112 μM for 0.5, 1, 3, or 6 h. After exposure, the supernatant was discarded, 100 μL of fresh RPMI media was added to each well and incubated at room temperature for 30 min. We added 100 μL of Steady-ATP Buffer into each well, put the plate on the shaker for 2 min, collected the mixture into Eppendorf tubes, and incubated them at room temperature for 10 min. We then read the luminescence, recorded RLU/s, and calculated for ATP concentrations for each treatment. A positive control of 20% DMSO was included. Each treatment was tested in triplicate.

### 2.11. Statistical Analysis

Data were analyzed using one-way ANOVA followed by Dunnett’s multiple comparisons test. Normality and homogeneity of variances were confirmed using the Shapiro–Wilk and Brown–Forsythe tests, respectively, in GraphPad Prism 10.4.2 (Boston, MA, USA), indicating that the assumptions for ANOVA were satisfied. Data were presented on graphs as means ± SD, with * *p* < 0.05, ** *p* < 0.01, *** *p* < 0.001, and **** *p* < 0.0001. Each treatment condition was measured in technical replicates within an experiment.

All statistical analyses were performed at a significance level of *p* < 0.05. We first used the Shapiro–Wilk test to confirm the normality of data distribution. The homogeneity of variances was checked with a Brown–Forsythe test. Since both were satisfied, we used one-way ANOVA followed by Dunnett’s multiple comparison tests on all data. Data were presented on graphs as means ± SD, with * *p* < 0.05, ** *p* < 0.01, *** *p* < 0.001, and **** *p* < 0.0001. All experiments were performed with three independent biological replicates, each measured using three or six technical replicates. Statistics were conducted using all measured values in GraphPad Prism. The relatively small sample size may limit statistical power, and results should be interpreted accordingly.

## 3. Results

### 3.1. PFOS Inhibited Cell Growth in HepG2 and THLE-2 Cells

**Figure 1:**
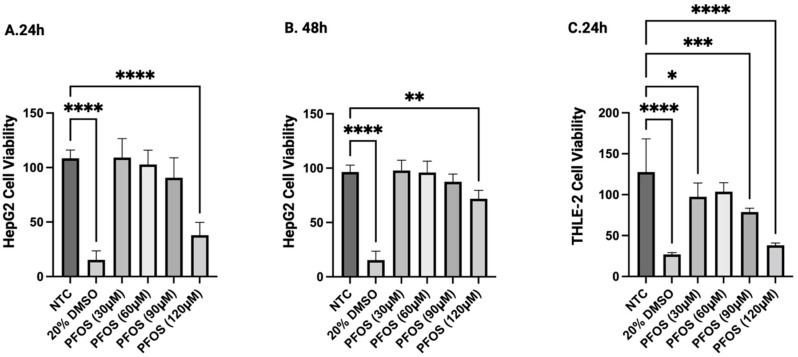
PFOS inhibited cell proliferation in HepG2 and THLE-2 cells in a dose-dependent manner. HepG2 and THLE-2 cells were exposed to 0, 30, 60, 90, or 120 μM PFOS, and 20% DMSO as positive control for 24 and 48 h. (**A**) HepG2 cell viability upon 24 h treatment. (**B**) HepG2 cell viability upon 48 h treatment. (**C**) THLE-2 cell viability upon 24 h treatment. Data were expressed as the means ± SD, *n* = 6, * *p* < 0.05, ** *p* < 0.01, *** *p* < 0.001, **** *p* < 0.0001, using GraphPad Prism.

We observed a dose-dependent decrease in the number of viable cells in both cell lines after 24 and 48 h of PFOS treatment. In HepG2, cell viability was slightly affected by PFOS from 30 μM to 90 μM but was significantly inhibited by PFOS at 120 μM after both 24 and 48 h (Figure 1A,B). In THLE-2 cells, it was shown that PFOS at all concentrations tested except for 60 μM remarkably hindered cell proliferation. Therefore, compared to the liver cancer cells, normalized liver cells showed a higher sensitivity to PFOS by approximately 50% (Figure 1C). Particularly, at the highest concentration of 120 μM, the most significant decrease in the cell viability of both cell lines was seen (**** *p* < 0.0001). Based on the results obtained from 24 h treatments, ${IC}_{50}$ values were determined using Prism GraphPad, with the concentrations of 112 μM and 93 μM for HepG2 and THLE-2, respectively, and these concentrations of PFOS were adopted for further biochemical assays (Table 1). Due to slow growth and high detachment rates in the THLE-2 cell line after each sub-culture, we decided to proceed to further experiments with HepG2 cells only.

### 3.2. PFOS Induced Morphological Changes in HepG2 Cells

**Figure 2:**
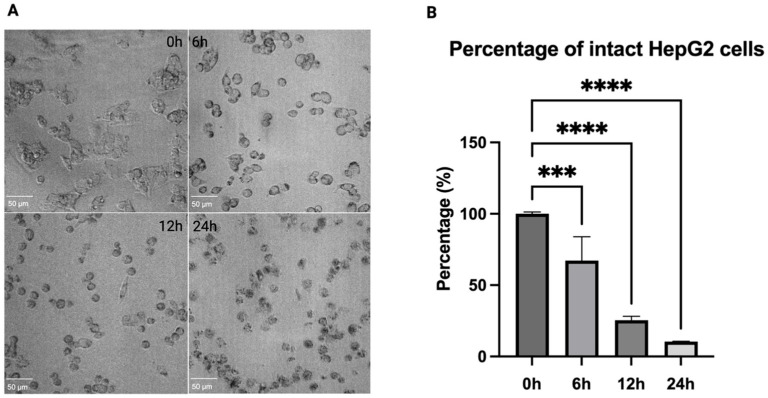
Time-dependent morphological changes in HepG2 cells and quantitation of intact cells after 112 μM PFOS exposure. (**A**) DIC images show progressive loss of normal morphology and attachment over time. (**B**) Quantitative analysis demonstrates a reduction in intact cells after 6, 12, and 24 h. Data are expressed as the means ± SD, *n* = 3, *** *p* < 0.001, **** *p* < 0.0001, using GraphPad Prism. Scale bar is 50 µm.

Differential interference contrast (DIC) microscopy revealed time-dependent morphological changes in PFOS-treated HepG2 cells (Figure 2A). At 0 h, cells exhibited normal polygonal morphology with intact attachment to the culture plate. Early morphological changes, including cell rounding and reduced cell spreading, started as early as 6 h of treatment. These changes became more noticeable at 12 h, with substantial loss of attachment, and decreased cell density. By 24 h, pronounced morphological disruption was observed, characterized by extensive cell detachment, membrane rupture, and the presence of floating cells and cellular debris. Consistent with these DIC observations, quantitative analysis demonstrated a reduction in the percentage of intact HepG2 cells from 100% at 0 h to 67.15 ± 16.79%, 25.38 ± 2.79%, and 10.42 ± 0.26% at 6, 12, and 24 h, respectively (Figure 2B). As a result, we decided to move forward with a 6 h PFOS treatment for further experiments.

### 3.3. PFOS-Induced Intracellular Reactive Oxygen Species (ROS) Formation in HepG2 Cells

**Figure 3:**
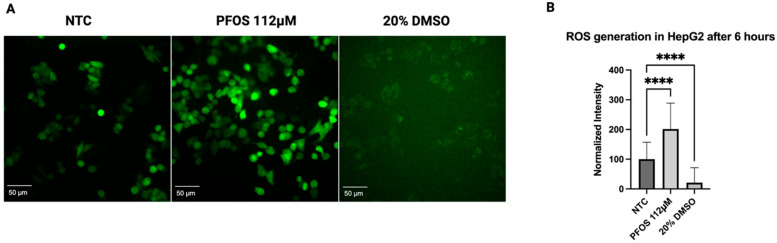
PFOS induced oxidative stress in HepG2 after 6 h. (**A**) Representative fluorescence microscopy images showed an elevation in ROS levels in HepG2 cells treated with 112 μM PFOS compared to non-treated control (NTC). (**B**) Quantitative analysis demonstrated a significant increase in ROS generation in PFOS-treated cells. Data are expressed as means ± SD, **** *p* < 0.0001, *n* = 3. Scale bar is 50 µm.

To assess whether the cell viability defects are in part due to an increased level of oxidative stress upon the PFOS exposure, we used DCFH-DA (2′,7′-dichlorofluorescein diacetate) detecting intracellular reactive oxygen species (ROS). Consistent with previous reports demonstrating that PFOS could induce ROS generation [38,39,45], we observed a stark increase in ROS in HepG2 cells, with the concentration of 112 μM of PFOS after 6 h (Figure 3A). Statistical data showcased an increase in the fluorescence intensity of oxidized DCF in PFOS-treated cells, approximately two-fold higher (201.66 ± 87.23%) when compared with the non-treated samples (Figure 3B). Dimethyl sulfoxide (DMSO) was included as a control.

### 3.4. PFOS Induces Early Apoptosis in HepG2 Cells

**Figure 4:**
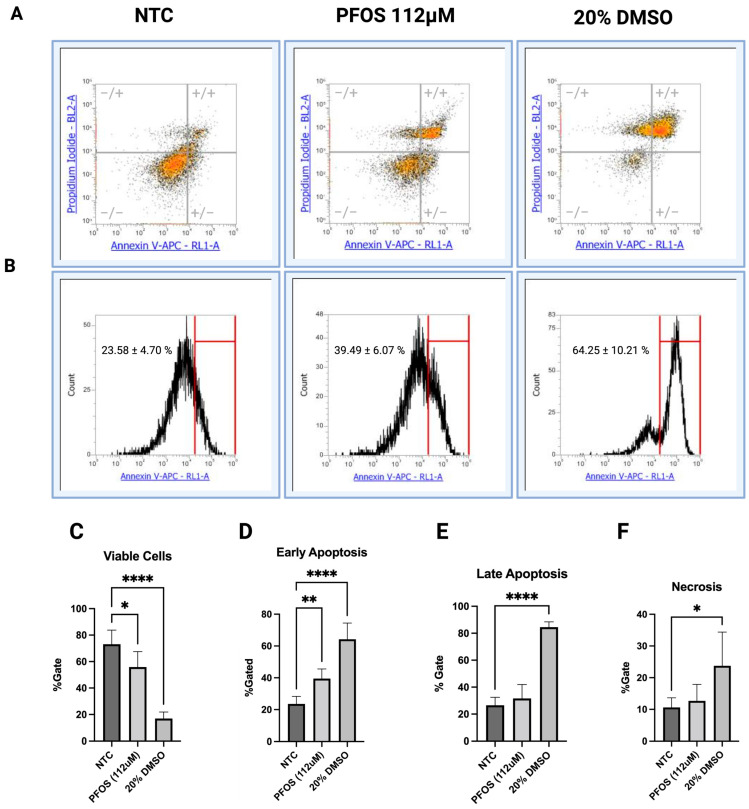
Increased early apoptotic levels in HepG2 cells upon 6 h PFOS exposure. (**A**) Representative flow cytometry dot plots showing distribution of HepG2 cells across quadrants based on Annexin V and propidium iodide (PI) staining. Quadrants represented viable (−/−), early apoptotic (+/−), late apoptotic (+/+), and necrotic (−/+) cell populations. (**B**) Representative histogram of Annexin V fluorescence intensity showing a bell-shape distribution. The H gate indicates the Annexin-V-positive population used for quantitative analysis of early apoptotic cells. Quantitative analysis demonstrated a significant decrease in viable cells (**C**), a significant increase in early apoptotic cells (**D**), and no remarkable changes in late apoptotic (**E**) or necrotic (**F**) cells after 6 h exposure to PFOS at 112 μM. (**C**–**F**) Asterisks indicate the significant differences between NTC- and PFOS-treated groups, and between NTC- and DMSO-treated groups. Data are expressed as means ± SD, * *p* < 0.05, ** *p* < 0.01, **** *p* < 0.0001, *n* = 3.

The observed decrease in cell viability, progressive loss of cell attachment, and increased levels of intracellular ROS upon PFOS exposure suggested the possible involvement of apoptotic processes in HepG2 cells. Although PFOS-induced apoptosis in HepG2 cells has been reported [37], the onset of early apoptotic events remains incompletely characterized. In our study, flow cytometry analysis revealed that HepG2 cells exhibited early apoptotic changes as early as 6 h after exposure to PFOS at 112 μM. Specifically, PFOS treatment resulted in the emergence of two distinct cell populations compared to NTC cells. These clusters corresponded to subpopulations of viable cells (Annexin V−/PI−), and apoptotic cells (Annexin V+) (Figure 4A). Gating analysis demonstrated a clear shift in population distribution, with 39.49 ± 6.07% of cells within the designated apoptotic gate compared to 23.58% ± 4.70 in NTC (Figure 4B). Consistent with the visualization, the proportion of viable cells decreased from 73.14% ± 10.61 in NTC to 55.87% ± 11.65 upon PFOS treatment (Figure 4C). In contrast, the percentage of early apoptotic cells significantly increased in PFOS-treated groups (39.49% ± 6.07) compared to 23.58% ± 4.70 in NTC (Figure 4D). No remarkable changes were observed in late apoptosis or necrosis between PFOS-treated and control groups (Figure 4E,F), indicating that PFOS primarily induced early apoptotic signaling at 6 h. DMSO was included as a control.

### 3.5. Transcriptomic Analysis Unravels Significant Differential Gene Expression in HepG2 Cells

#### 3.5.1. Differentially Expressed Genes (DEGs)

**Figure 5:**
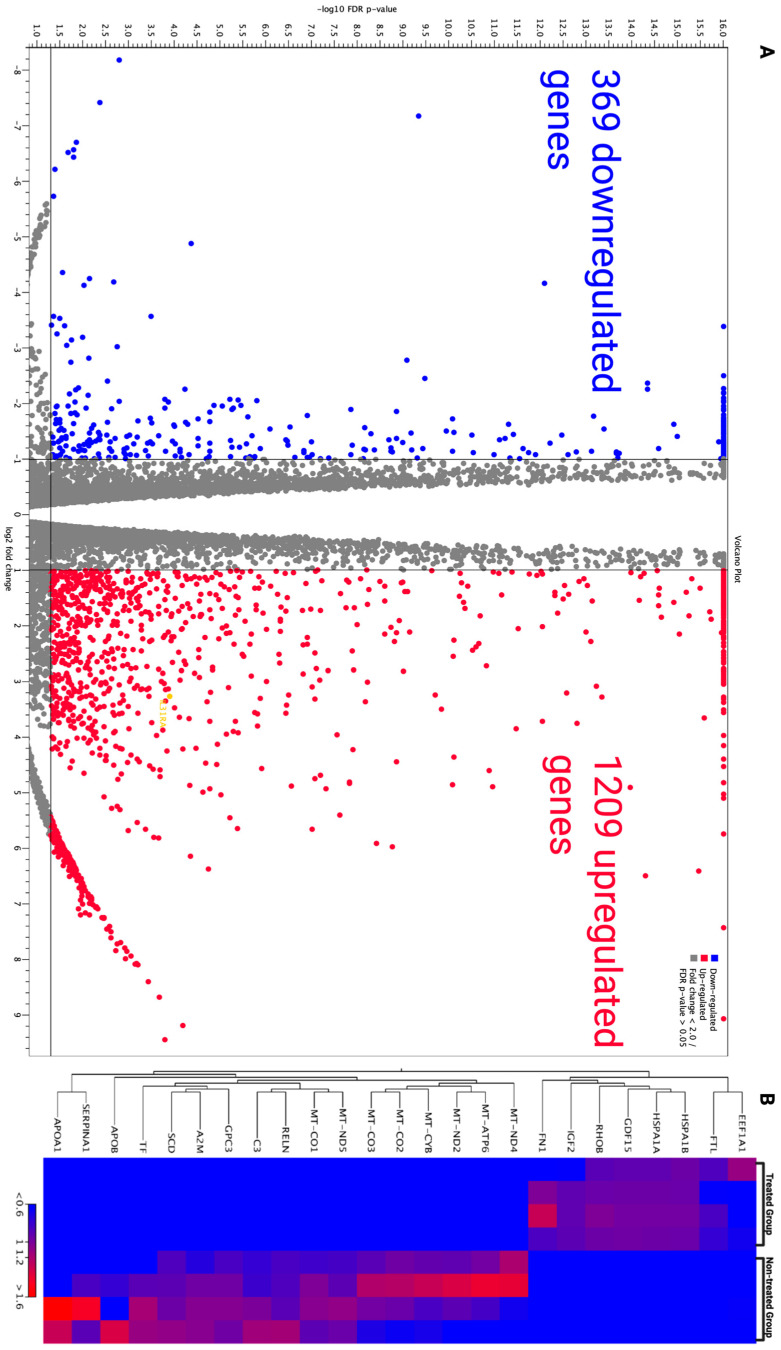
Down and upregulated gene transcripts in HepG2 cells after 6 h exposure with PFOS. (**A**) Volcano plot demonstrated 369 downregulated and 1209 upregulated genes, ${log}_{2}FC$ ≤ −1.0 for downregulation and ${log}_{2}FC$ ≥ 1.0 for upregulation, and FDR *p*-value ≤ 0.05. (**B**) Heat map showing differential expression of selected genes in HepG2 upon 6 h PFOS exposure. Rows represented genes, and columns represented samples from PFOS-treated and non-treated groups. Gene expression levels were represented as ${log}_{2}FC$, with the color scale ranging from blue (downregulated) to red (upregulated).

To predict defective or altered biological/molecular pathways in a comprehensive manner in response to PFOS exposure, we conducted RNA sequencing analysis. We identified 1209 upregulated and 369 downregulated gene transcripts in HepG2 cells upon 6 h exposure to PFOS at 112 μM, as shown in a volcano chart by plotting −log_10_ (FDR) on the y-axis and log_2_ (fold change) on the x-axis (Figure 5A). Differentially expressed genes (DEGs) were identified using a cutoff of ${log}_{2}fold\;change$ ≥ 1.0 for upregulated genes and ${log}_{2}FC$ ≤ −1.0 for downregulated genes, with a false discovery rate (FDR)-adjusted *p*-value ≤ 0.05. In treated groups, hierarchy clusters showcased the significant downregulation of mitochondrial genes such as *MT-ND4, MT-ATP6, MT-ND2, MT-CO2*, and *MT-CO3*, and remarkable upregulation of heat shock proteins such as *HSP1A* and *HSP1B* (Figure 5B).

**Figure 6:**
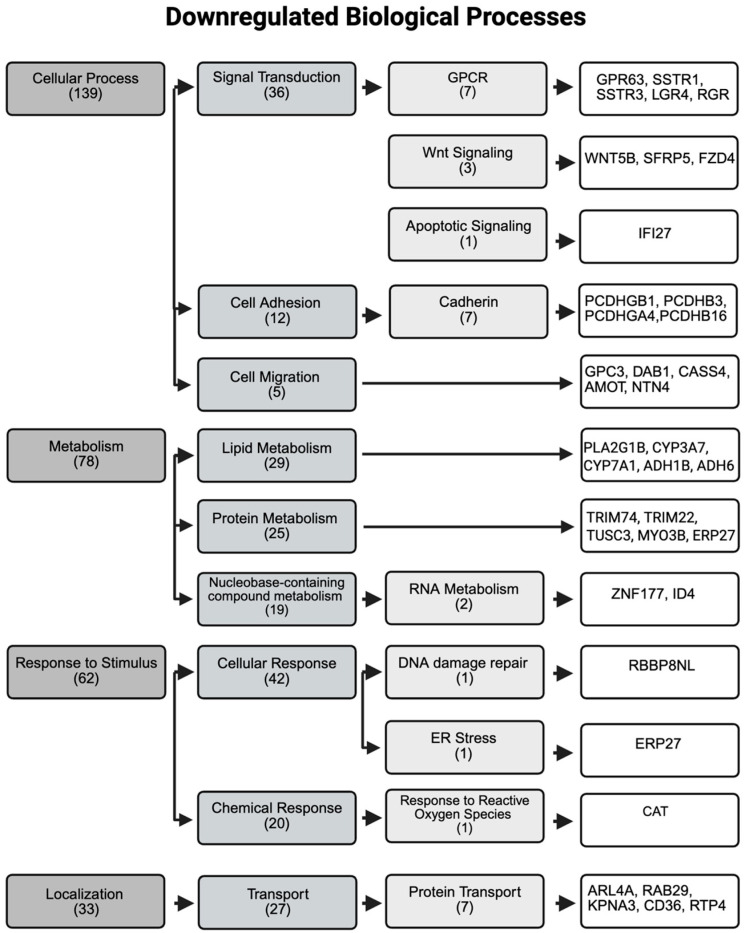
Major downregulated biological processes with key genes associated. Data were obtained from Panther Ontology, with numerical numbers indicating the total affected genes in each category and subcategory. Created in BioRender. Tran. (2026). https://BioRender.com.

**Figure 7:**
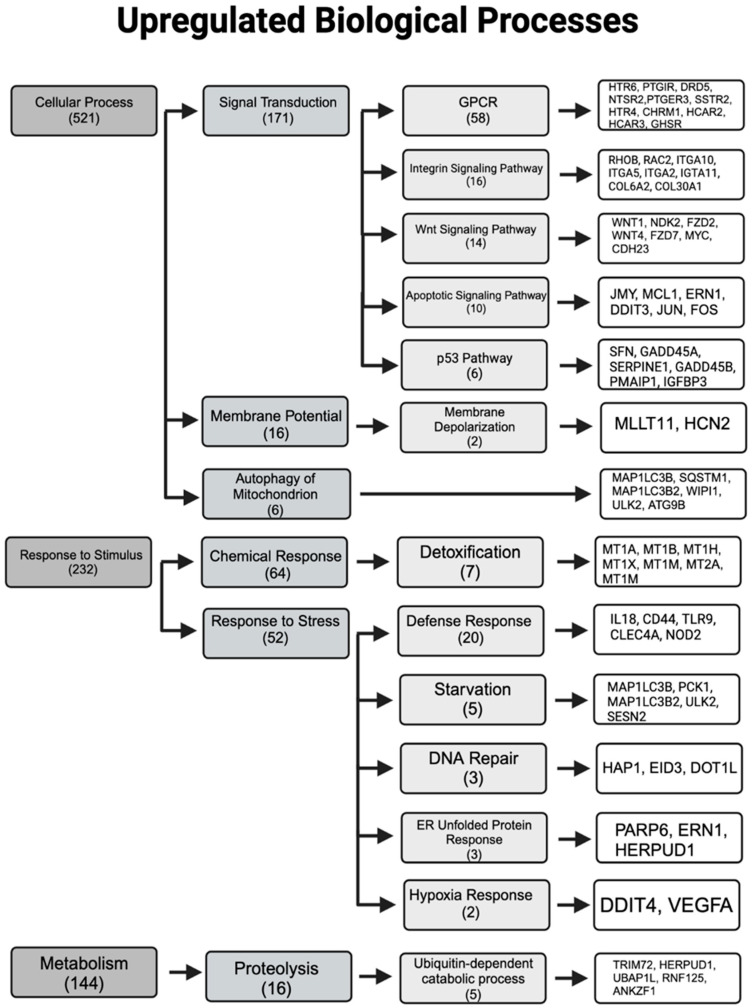
Major upregulated biological processes with key genes associated. Data were obtained from Panther Ontology, with numerical numbers indicating the total affected genes in each category and subcategory. Created in BioRender. Tran. (2026). https://BioRender.com.

DEGs are critical indicators of alteration in biological activity of cells, and therefore, we created a diagram highlighting the significantly impacted processes along with key genes associated with each process (Figure 6 and Figure 7). The top-level category with the largest number of affected genes was cellular processes, comprising 139 downregulated and 521 upregulated genes. Within this category, genes involved in lateral cell adhesion (*PCDHGB1*, *PCDHB3*, *PCDHGA4*, and *PCDHB16*) were downregulated (Figure 6) and integrin genes were upregulated (*ITGA11*, *ITGA5*, *ITGAX*, *ITGA2*, and *ITGA10*) (Figure 7). The apoptotic signaling pathway was significantly upregulated, with 14 impacted genes including *JMY*, *MCL1*, *DDIT3*, *JUN*, and *FOS* (Figure 7). Metabolic processes exhibited substantial numbers of both down and upregulated gene transcripts (Figure 6 and Figure 7). Notably, genes related to detoxification (*MT1A*, *MT1B*, *MT1H*, *MT1M*, and *CYP7A1*) and defense responses (*IL18*, *CD44*, *TLR9*, and *NOD2*) were remarkably upregulated (Figure 7), whereas genes involved in DNA damage repair (*RBBP8NL*) and response to ROS (*CAT*) were largely downregulated (Figure 6).

#### 3.5.2. Downregulated Genes Associated with Oxidative Phosphorylation

**Figure 8:**
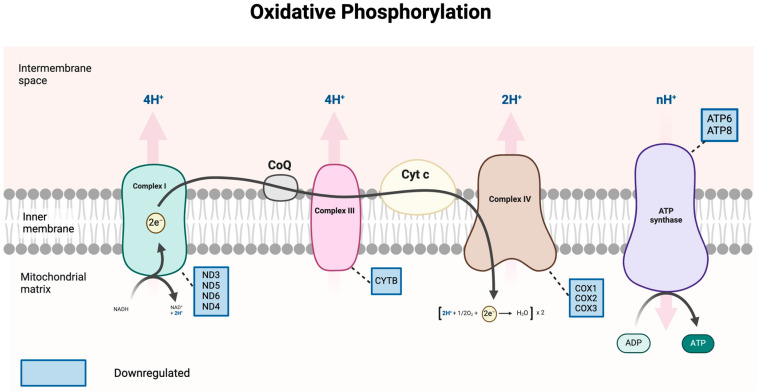
Downregulated genes functionally associated with mitochondrial protein complexes at the inner membrane of mitochondria. Complex I (NADH dehydrogenase) pumps H^+^ into the intermembrane space. *ND* genes downregulated. Complex III (cytochrome ${bc}_{1}$) transfers electrons to cytochrome c (Cyt c). *CYTB*, a component of Complex III, was downregulated in response to PFOS exposure. Three *COX* genes for Complex IV (cytochrome c oxidase) were also downregulated. A couple of genes for ATP synthase were downregulated. Created in BioRender. Tran. (2026). https://BioRender.com.

We discovered a trend of downregulation of gene transcripts associated with the oxidative phosphorylation pathway. Specifically, upon exposure to PFOS (112 μM) after 6 h, 10 genes involved in the mitochondrial electron transport chain were significantly downregulated: four genes for complex I (*ND3*, *ND5*, *ND6*, and *ND4*), one gene for complex III (*CYTB*), three genes for complex IV (*COX1*, *COX2*, and *COX3*), and two genes for ATP synthase (*ATP6* and *ATP8*) (Figure 8). Table 2 summarizes the gene names associated with each protein complex, together with their fold changes.

#### 3.5.3. Dynamics of Cell Adhesion Molecules (CAMs) upon PFOS Exposure

**Figure 9:**
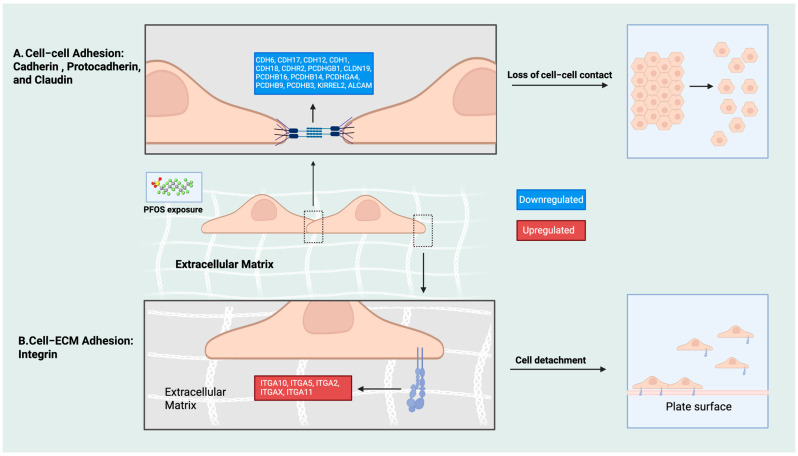
Dynamics of cell adhesion molecules (CAMs) upon PFOS exposure at 112 μM after 6 h. (**A**) Disruption of cell–cell adhesion in HepG2 cells. Cadherin, protocadherin, and claudin gene expression levels were downregulated. Cells exhibited reduced cohesion and separation from neighboring cells. (**B**) Disruption of cell–extracellular matrix (ECM) in HepG2 cells. Integrin genes were upregulated. A substantial number of cells detached from the plate surface. Created in BioRender. Tran. (2026). https://BioRender.com.

Upon PFOS exposure at 112 μM, we discovered that the dynamics among cell adhesion molecules (CAMs) were significantly altered after 6 h. In brief, cell–cell adhesion proteins, including cadherin, protocadherin, and claudin, were significantly downregulated (Figure 9A). Since cell–cell adhesion is required to maintain connections between neighboring cells [46], we concluded that the loss of cell–cell contacts (Figure 2A) might be due to a dissociation of previously cohesive cell clusters upon the downregulation of adhesion genes. In contrast, cell–extracellular matrix (ECM) binding proteins such as integrins were upregulated (Figure 9B). The altered level of integrins can account for the disruption of cell–ECM adhesion, which led to dysregulation of the cell shape and resulted in a higher chance of cell detachment (Figure 2B). Table 3 summarizes the gene names associated with CAM dynamics.

#### 3.5.4. RT-qPCR

**Figure 10:**
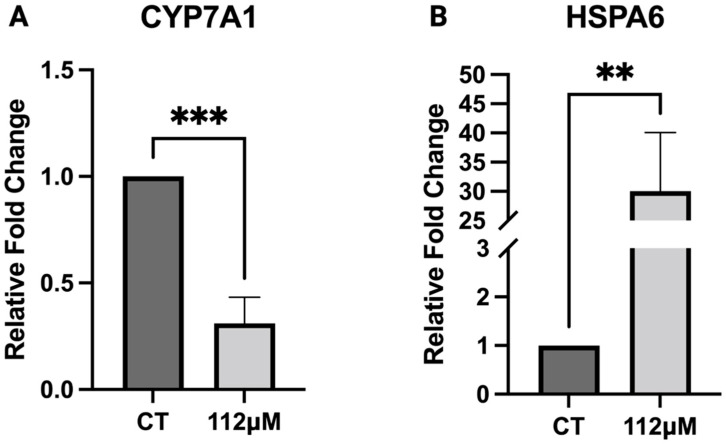
Validation of RNA-seq result by RT-qPCR. RT-qPCR was used to determine the differential expression of genes involved in xenobiotic metabolism and protein homeostasis including (**A**) Cholesterol 7a-hydroxylase (*CYP7A1*) and (**B**) Heat shock protein Family A (Hsp70) Member 6 (*HSPA6*) in HepG2 liver cancer cells upon 6 h PFOS exposure as compared to the control group. Data are presented as means ± SD, ** *p* < 0.01, *** *p* < 0.001, *n* = 3 for each group.

The accuracy and reproducibility of the RNA-seq data were confirmed by the RT-qPCR validation using Succinate Dehydrogenase Complex Subunit A (*SDHA*) as a non-differentially expressed reference gene. Two key DEGs were selected including *CYP7A1* and *HSPA6*. *CYP7A1*, a cytochrome P450 gene, is a rate-limiting enzyme implicated in bile acid synthesis and xenobiotic detoxification [47,48]. The relative fold change (FC) of *CYP7A1* was reduced to 0.31 ± 0.12 compared to the control (FC = 1.0), a statistically significant reduction in gene expression (Figure 10A). *HSPA6* is a heat shock protein, facilitating protein folding, assembly, and quality control [49,50]. We observed a remarkable upregulation in the relative fold change in HSPA6, approximately a 30-fold increase (30.1 ± 10.04) compared to the control (Figure 10B). Forward and reverse primer sequences used for the PCR reactions are provided in Table 4.

#### 3.5.5. ATP Levels Are Significantly Reduced After 3 h Exposure to PFOS

**Figure 11:**
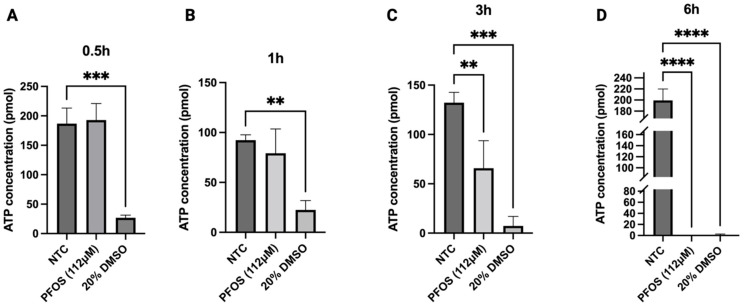
ATP production in HepG2 cells upon PFOS exposure at 112 μM for 0.5, 1, 3, and 6 h. (**A**) No significant differences were observed between treated and control groups after 0.5 h. (**B**) No significant differences were observed between treated and control groups after 1 h. (**C**) ATP levels were significantly reduced after 3 h compared to NTC. (**D**) ATP production remarkably decreased at 6 h. Data are expressed as means ± SD, ** *p* < 0.01, *** *p* < 0.001, **** *p* < 0.0001, *n* = 3.

Sustained ROS accumulation could result in mitochondrial dysfunction [51]. Our transcriptomic analysis predicts the disruption in the ETC, which is a hallmark feature of homeostatic imbalance (Figure 8). Furthermore, multiple genes implicated in the activity of ETC protein complexes were significantly downregulated, especially in ATP synthase complex (Table 2), and therefore, we conducted an assay to measure ATP production rate in response to PFOS. Figure 11 illustrates ATP production in HepG2 cells after 0.5, 1, 3, and 6 h exposure to PFOS. No significant differences in ATP levels were observed between PFOS-treated and control groups after 0.5 h and 1 h (Figure 11A,B). However, upon 3 h of PFOS exposure, ATP levels in the treated group were significantly reduced to 65.77 ± 27.92 pMol compared to the NTC group (132.22 ± 10.44 pMol) (Figure 11C). By 6 h, ATP production remarkably decreased to 0.091 ± 0.01 pMol in the treated group (Figure 11D). DMSO was included as a control.

## 4. Discussion

**Figure 12:**
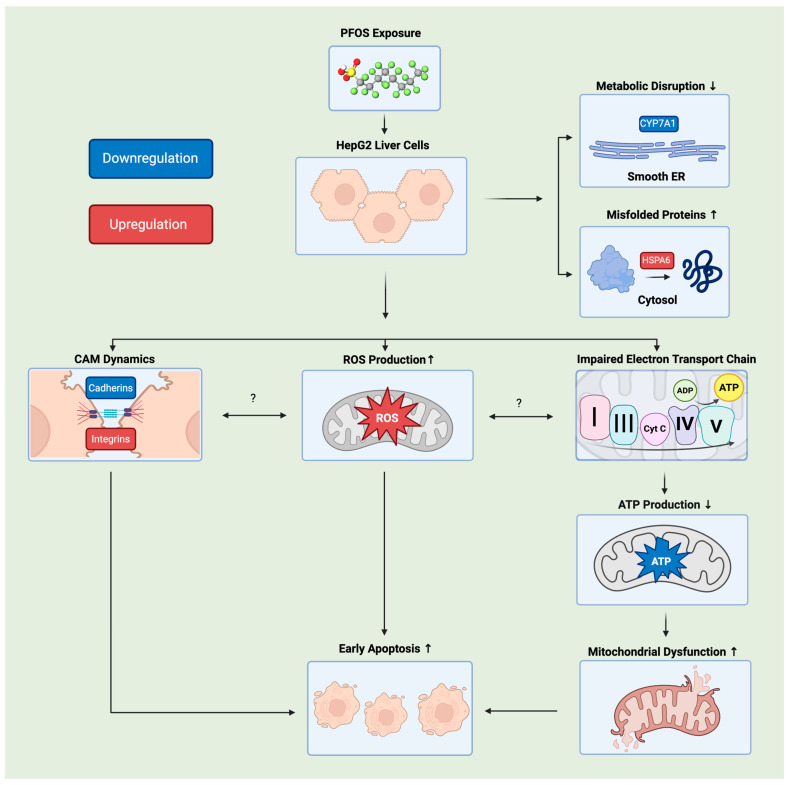
Schematic diagram showcases complex relationships among different biological elements in HepG2 cells upon PFOS exposure. Upon PFOS exposure, intracellular reactive oxygen species (ROS) levels increased, electron transport chain (ETC) was impaired, and cell adhesion molecule (CAM) dynamics were altered. It was followed by a reduction in ATP production, metabolic disruption, and an increase in misfolded proteins. Eventually, these changes contributed to the onset of early apoptosis in HepG2 cells. Created in BioRender. Tran. (2026). https://BioRender.com. The arrows represented up- or downregulation of specific processes. The question marks are needed for the unknown relationship among those pathways.

Produced since the 1950s, PFOS has been one of the most common persistent contaminants that poses significant risks to the human health [10,52]. Human populations can come into contact with PFOS via multiple pathways such as environmental, occupational or oral exposures. Epidemiological studies have shown that PFOS is detected in blood, serum, and kidneys, but mainly in the human liver. Although the liver plays an essential function in metabolism and xenobiotic detoxification [53,54,55], very few studies have thoroughly investigated PFOS hepatotoxicity. Similar trends in ROS elevation, mitochondrial dysfunction, and apoptosis activation have been observed in other liver models, including rodent and fish hepatocytes, as well as human liver cell lines, suggesting the potential cellular stress response to PFOS exposure [56,57,58]. To the best of our knowledge, the present study extends current understanding of PFOS toxicity by demonstrating that, as early as 6 h post-exposure, PFOS simultaneously disrupts cell adhesion dynamics, causes mitochondrial dysfunction, and induces early apoptosis in HepG2 liver cancer cells (Figure 12).

Cell adhesion is known to play a key role in the development and maintenance of cell structures, including cell–extracellular matrix (ECM) and cell–cell adhesion [46]. Transmembrane proteins, such as integrins, are essential in the binding of ECM and connecting to the cellular cytoskeleton, modulating cell signaling across the plasma membrane, and regulating the shape of cells [59]. Cell–cell adhesion proteins, such as cadherins, regulate cell–cell contacts to maintain the connections between neighboring cells [46]. Since cell adhesion is essential for maintaining cell structure and mediating cell communication, alterations in adhesion dynamics can disrupt cell differentiation, apoptosis, and proliferation, ultimately influencing cell fate [59,60]. In our study, we observed the changes in HepG2 cell morphology over time, with visual confirmation of cell detachment and loss of cell–cell contact after a 6 h incubation with PFOS (Figure 2). Furthermore, as the expression of CAMs, including integrin, cadherin, and protocadherin, was altered, we propose that PFOS-induced disruption of cell adhesion represents an early cellular response, possibly caused in part by a significantly reduced level of mitochondrial-based ATP production after a 3 h incubation of HepG2 with PFOS (Figure 11), for ATP is required for stabilizing several integrin-linked kinases [61]. However, there is insufficient evidence that an elevation of ROS (Figure 3) is directly implicated in inducing damage of mitochondria and the subsequent low level of ATP production. A recent report demonstrated that PFOA affects integrins and reduces cell adhesion in a lung cancer cell (A549) [62]. However, to our knowledge, no previous studies have reported such similar impairment in HepG2 cells affected by PFOS. With our observation of downregulation of several integrin and cadherin genes, we propose that not only cell-to-matrix but also the adherence junction and lateral cell-to-cell junction are disrupted within 6 h of PFOS treatment.

It is well-known that the overproduction of reactive oxygen species (ROS), byproducts of cellular metabolism, is a primary indicator of changes in cellular homeostasis [63,64]. Our data demonstrated a remarkable elevation of ROS by 2-fold in HepG2 cells following 6 h of PFOS exposure (Figure 3). We also found other studies that reported a similar trend. A previous study by Eriksen, et al. in 2010 showed that PFOS exposure could induce ROS generation by 1.25-fold in HepG2 liver cells after 3 h [39]. Amstutz et al., (2022) also measured ROS generation and showed that, upon PFOS treatment, ${AUC}_{ROS}$ was elevated to 1.4 ± 0.2 and 3.9 ± 0.8 after 3 and 24 h, respectively, relative to the control group (control = 1) [38]. Both studies used the DCFH-DA assay and quantified ROS using an area-under-the-curve (AUC) approach, which integrates ROS levels over time and reflects the cumulative oxidative burden. Hu et al. (2009) also reported significant ROS generation after 5, 10, and 15 h of PFOS exposure [45]. Other research studies produced a mix of results, but the majority reported significant changes in ROS levels after 24 h of exposure [65,66,67]. Although most studies have applied similar methods to measure reactive oxygen species (ROS) levels, these assays primarily detect total intracellular ROS rather than individual oxidant species. Since ROS comprise a wide variety of oxidant molecules with different biological functions and properties [68], future studies should focus on identifying the predominant ROS species to better define the oxidative stress mechanisms underlying PFOS exposure. Several in vivo studies have investigated the toxic effects of PFOS exposure on hepatic mitochondria. In a Sprague–Dawley rat liver model, transmission electron microscopy (TEM) analyses revealed remarkable mitochondrial structural damage, such as lost cristae, swollen vacuole structure, and disturbed rough endoplasmic reticulum (ER) [19]. A transcriptomic study in 2025 on *Carassius auratus,* a fish liver model, reported significant disruption in OXPHOS-related pathways. Several ATP synthase-associated genes were upregulated, suggesting a potential disruption in ATP production [25]. Despite these clear morphological and gene expression alterations, both studies did not assess mitochondrial functions directly, limiting conclusions to qualitative observations and unknown mechanisms underlying PFOS toxicity. Additionally, the species-specific variability in mitochondrial structure and bioenergetic function have been a challenge to the translation of results from animal studies to human physiology [69]. Our current data revealed multiple downregulated mitochondrial genes implicated in the activity of the ETC (Table 2), accompanied by a significant reduction in ATP levels as early as at 3 h. Given that reduced ETC gene expression can promote electron or proton leakage and affect the mitochondrial health [70], our findings suggested that PFOS exposure could trigger mitochondrial dysfunction. Furthermore, there is a well-known bidirectional relationship between ROS generation and mitochondrial dysfunction [71]. However, we have yet to know whether the impairment of ETC could be caused by elevated ROS levels or whether they could happen simultaneously. In the future, investigations could be made to assess this relationship.

Research has shown that elevated ROS and mitochondrial dysfunction are commonly observed during the early stages of apoptosis, suggesting the initiation of the programmed cell death [72]. Early apoptosis is a process when a cell is committed to die but their membrane remains intact [73]. Phosphatidylserine (PS) will be externalized on the outer layers of the plasma membrane, recognized by Annexin V, a recombinant PS-binding protein [73,74]. Morphology of early apoptotic cells is characterized by distinct changes such as cell shrinkage, dense cytoplasm, and tightly-packed organelles [73,75]. Determining the timing of early apoptosis is critical for distinguishing initial toxic events from downstream cellular collapse and for identifying mechanisms underlying irreversible cell death. Several studies have suggested PFOS-induced apoptotic responses in HepG2 cells. However, few discuss the onset of early apoptosis. In a study by Yao et al. (2016), PFOS exposure in HepG2 was assessed using Annexin V/PI staining. However, early apoptotic signals were detected after 24 h of incubation with PFOS 200 μM. The study also reported that PFOS-treated cells were observed with accumulations of giant mitochondria [76]. The higher treatment concentration and longer incubation time were used to assess the PFOS-induced autophagy-dependent apoptosis in this study; therefore, it yielded different outcomes, including Caspase-3 upregulation [76]. Flow cytometry analysis by Hu et al. (2009) also reported apoptosis in HepG2 upon PFOS exposure, however, they focused on late apoptosis and necrosis. Overall, the study reported concentration-dependent cell death, with evidence of increased Caspase-9 activity and downregulation of Bcl-2 after 48 h of PFOS exposure. The study primarily reported total apoptotic levels at a later time point, and discussed the abnormal expressions of key apoptosis markers [45]. While previous studies primarily examined PFOS-induced apoptosis at higher concentrations and longer exposure periods, our present study focuses on earlier time points and lower exposure levels. Therefore, we uniquely proposed the first detectable occurrence of early apoptosis, revealing that PFOS could initiate apoptotic signals prior to extensive cell death as early as at 6 h. It could be a triggering event following early mitochondrial dysfunction.

Cholesterol 7α-hydroxylase (CYP7A1), a member of cytochrome P450 family, is the key rate-limiting enzyme that plays an important role in regulation of bile acid synthesis in hepatocytes [47,48]. Our finding suggested that PFOS induced changes implicated in bile acid/cholesterol homeostasis, as evidenced by a decrease in CYP7A1 mRNA level (Figure 10A). A study by Behr et al. (2020) using HepaRG hepatoma cells also observed a reduction in CYP7A1 in both protein and gene expression levels upon PFOS exposure, showcasing impairments in bile acid synthesis [77]. Transcriptomic analysis on primary human hepatocytes from liver explants suggested that CYP7A1 downregulation could contribute to liver necrosis or cell death [78]. Since bile acid synthesis plays an important role in hepatic metabolism, we also suggested that the changes in CYP7A1 level could be one of the hallmarks in transcriptomic alterations in HepG2 cells upon PFOS exposure.

Heat shock protein Family A (Hsp70) Member 6 (HSPA6), a molecular chaperone, plays an essential role in maintaining cellular protein homeostasis, facilitating protein folding, assembly, membrane translocation, and quality control of proteins [49,50]. In our study, we observed a significant upregulation in gene expression of HSPA6. Currently, it was reported that a high level of HSPA6 might be associated with the recurrence of human hepatocellular carcinoma (HCC) [79]. In cancer and other conditions, its expression has been linked to cell survival, tumorigenesis, tumor progression, and response to cellular stress [49]. Although there are no previous studies linking PFOS exposure to HSPA6 expression, the gene belongs to the inducible Hsp70 family, which is typically upregulated upon cellular stress conditions to assist in protein folding and prevent proteotoxic damage [80]. Therefore, our finding suggested that changes in HSPA6 level may reflect its role in responding to PFOS-mediated protein unfolding.

In summary, this study investigated the toxicological effects of perfluorooctane sulfonate (PFOS) using HepG2 cells, a widely used model for studying human liver biology. While this cell line does not fully recapitulate the heterogeneity of liver tumors, our results provide important mechanistic insights into PFOS-induced hepatocellular responses. Although the data from our study were obtained at a relatively higher concentration compared to average human exposures, we predict that the knowledge gained from this study might be helpful for PFOS risk assessment given its persistence and bioaccumulative nature. Further studies using animal models and organoid systems are needed to validate these findings.

## Figures and Tables

**Table 1:** ${IC}_{50}$ and ${IC}_{20}$ values. ${IC}_{50}$ and ${IC}_{20}$ values were calculated based on the results obtained from 24 h treatments using GraphPad Prism.

Cell Lines	${IC}_{50}$ Values (μM)	${IC}_{20}$ Values (μM)
HepG2	112 ± 2.28	97 ± 1.46
THLE-2	93 ± 0.57	86 ± 4.70

**Table 2:** List of DEGs associated with oxidative phosphorylation.

	GeneCards Symbol/Gene Names	Gene Descriptions	Fold Change
Complex I	*MT-ND3/ND3*	Mitochondrially Encoded NADH:Ubiquinone Oxidoreductase Core Subunit 3	−2.11
	*MT-ND5/ND5*	Mitochondrially Encoded NADH:Ubiquinone Oxidoreductase Core Subunit 5	−2.21
	*MT-ND6/ND6*	Mitochondrially Encoded NADH:Ubiquinone Oxidoreductase Core Subunit 6	−2.97
	*MT-ND4/ND4*	Mitochondrially Encoded NADH:Ubiquinone Oxidoreductase Core Subunit 4	−1.69
Complex III	*MT-CYB/CYTB*	Mitochondrially Encoded Cytochrome B	−2.01
Complex IV	*MT-CO1/COX1*	Mitochondrially Encoded Cytochrome C Oxidase I	−3.01
	*MT-CO2/COX2*	Mitochondrially Encoded Cytochrome C Oxidase II	−2.04
	*MT-CO3/COX3*	Mitochondrially Encoded Cytochrome C Oxidase III	−2.06
ATP synthase	*MT-ATP6/ATP6*	Mitochondrially Encoded ATP Synthase Membrane Subunit 6	−1.77
	*MT-ATP8/ATP8*	Mitochondrially Encoded ATP Synthase Membrane Subunit 8	−1.85

**Table 3:** List of DEGs associated with cell adhesion.

Adhesion Types	Gene Names	Gene Description	Key Roles	Fold Change
Cell–cell Adhesion	*CDH6*	Cadherin 6	Cell differentiation, morphogenesis	11.11
	*CDH17*	Cadherin 17	Morphological organization of liver	47.38
	*CDH12*	Cadherin 12	Neuronal development	−14.34
	*CDH1*	Cadherin 1	Proliferation, invasion, metastasis	−1.09
	*CDH18*	Cadherin 18	Synaptic adhesion, axon outgrowth and guidance	−8.09
	*CDHR2*	Cadherin Related Family Member 2	Microvilli and epithelial brush border differentiation	−1.23
	*PCDHGB1*	Protocadherin Gamma Subfamily B,1	Establishment and function of specific cell–cell connections in the brain	−2.49
	*PCDHB16*	Protocadherin Beta 16	Cell–cell connections	−3.57
	*PCDHB14*	Protocadherin Beta 14	Cell–cell connections	−2.03
	*PCDHGA4*	Protocadherin Gamma Subfamily A,4	Cell–cell connections	−3.09
	*PCDHB9*	Protocadherin Beta 9	Cell–cell connections	−3.54
	*PCDHB3*	Protocadherin Beta 3	Cell–cell connections	−2.57
	*CLDN19*	Claudin 19	Tight junction association	−2.84
	*KIRREL2*	Kirre-Like Nephrin Family Adhesion Molecule 2	Adhere junction association	−2.32
	*ALCAM*	Activated Leukocyte Cell Adhesion Molecule	Cell migration	−2.06
Cell–ECM Adhesion	*ITGA10*	Integrin Subunit Alpha 10	Cell surface-mediated signaling	4.80
	*ITGA5*	Integrin Subunit Alpha 5	Cell surface-mediated signaling	2.32
	*ITGA2*	Integrin Subunit Alpha 2	Adhesion of platelets to the ECM	2.16
	*ITGAX*	Integrin Subunit Alpha X	Adherence of neutrophils and monocytes mediation	2.97
	*ITGA11*	Integrin Subunit Alpha 11	Adhesion of muscle tissue to the ECM	5.31

**Table 4:** Primer sequences for selected genes.

Gene Names	Primers
*CYP7A1*	Forward Sequence: 5′-TAC CAT AAG GTG TTG TGC CAC G-3′
	Reverse Sequence: 5′-CTT CTG TGC CCA AAT GCC TTC-3′
*HSPA6*	Forward Sequence: 5′-TGC TCA GAC CTC TTC CGC AG-3′
	Reverse Sequence: 5′-GCC TTG TCC AGC TTG GCA TC-3′
*SDHA*	Forward Sequence: 5′-AAC ACG GAC CTG GTG GAG AC-3′
	Reverse Sequence: 5′-TGG TGA GAA GGC CCA CCT TG-3′

## Data Availability

The original contributions presented in this study are included in the article/Supplementary Material. Further inquiries can be directed to the corresponding author.

## Supplementary Materials

The following supporting information can be downloaded at: https://www.mdpi.com/article/10.3390/jox16020065/s1, Figure S1: HepG2 morphology at h = 0; Figure S2: HepG2 morphology at h = 6; Figure S3: HepG2 morphology at h = 12; Figure S4: HepG2 morphology at h = 24; Figure S5: ROS generation in non-treated HepG2 cells; Figure S6: ROS generation in PFOS-treated HepG2 cells; Figure S7: ROS generation in 20% DMSO-treated HepG2 cells.
