# Supplementary material for: Perfluorooctane Sulfonate (PFOS) Disrupts Mitochondrial Activity and Cell Adhesion in Liver Cells
Source: J Xenobiot. 2026 Apr 13;16(2):65. doi: 10.3390/jox16020065 (PMC13118017; doi:10.3390/jox16020065)
Supplement: Supplementary file 1 [file jox-16-00065-s001.zip › jox-4179901-supplementary.pdf]

Journal of Xenobiotics

Supplementary Materials: Perfluorooctane Sulfonate (PFOS) Disrupts Mitochondrial Activity and Cell Adhesion in Liver Cells

Phuong D. Tran and Kyoungtae Kim

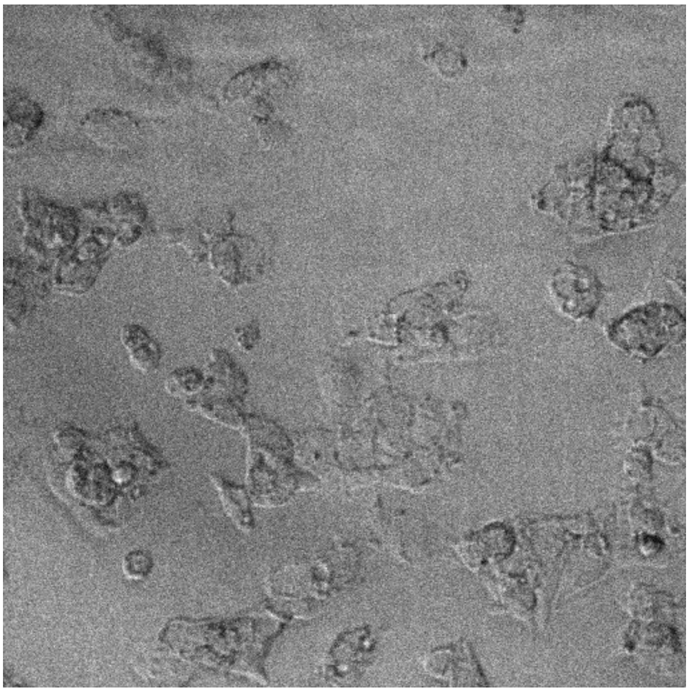

Figure S1: HepG2 morphology at h = 0 (Supplementary for figure 2A)

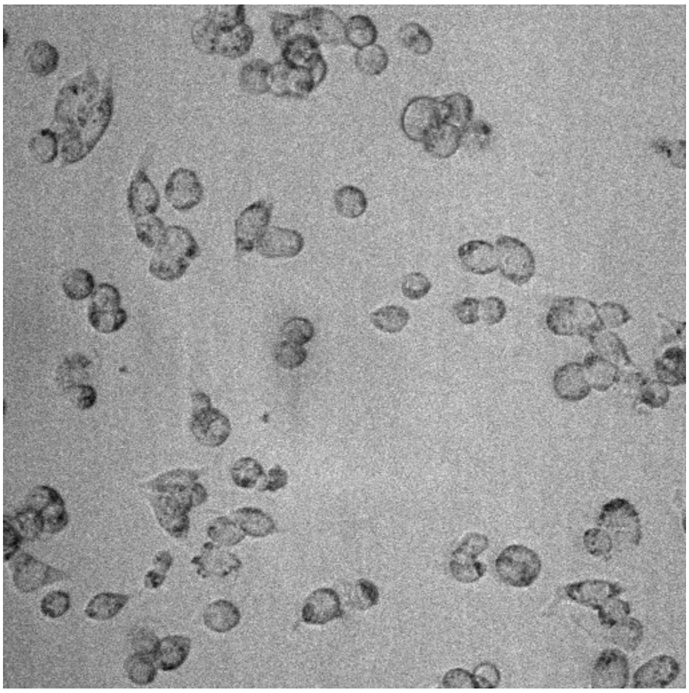

Figure S2: HepG2 morphology at h = 6 (Supplementary for figure 2A)

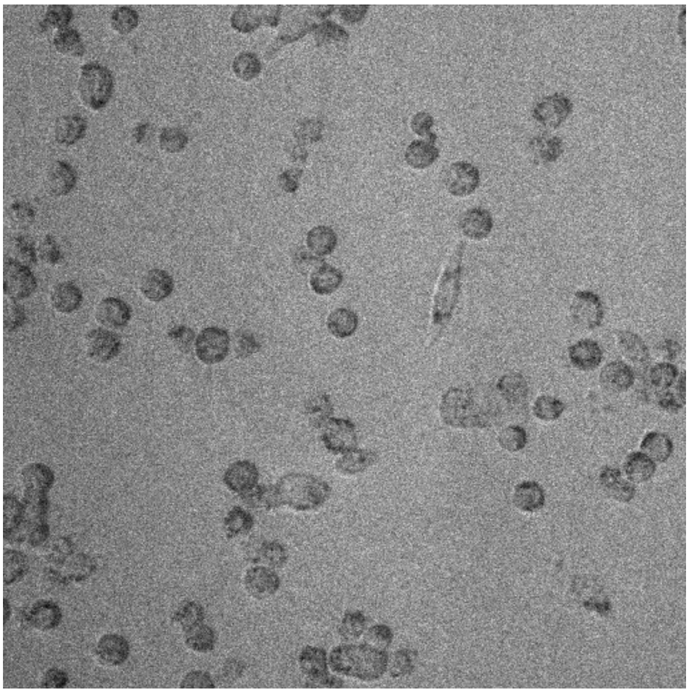

Figure S3: HepG2 morphology at h = 12 (Supplementary for figure 2A)

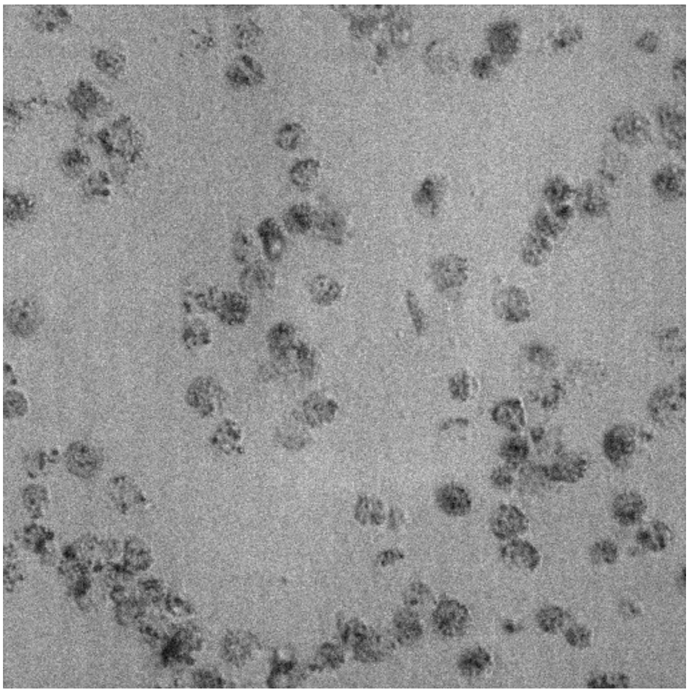

Figure S4: HepG2 morphology at h = 24 (Supplementary for figure 2A)

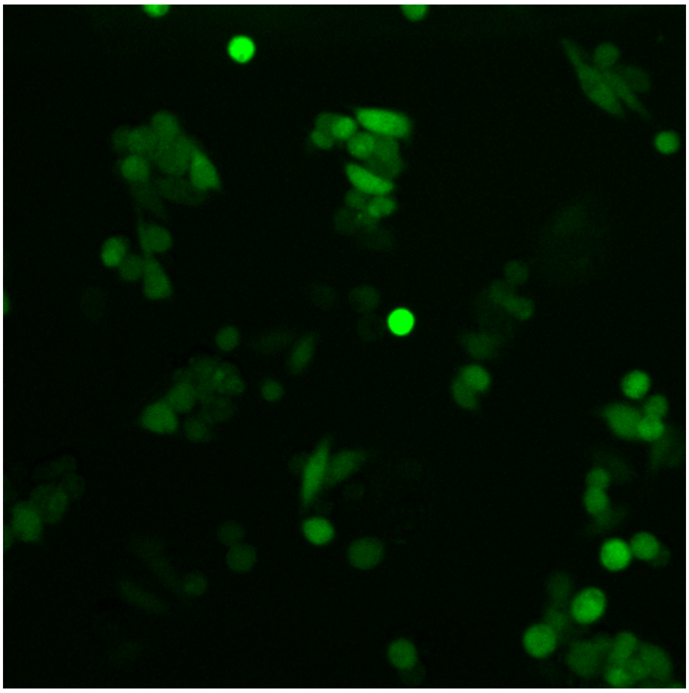

Figure S5: ROS generation in non-treated HepG2 cells (Supplementary for figure 3A)

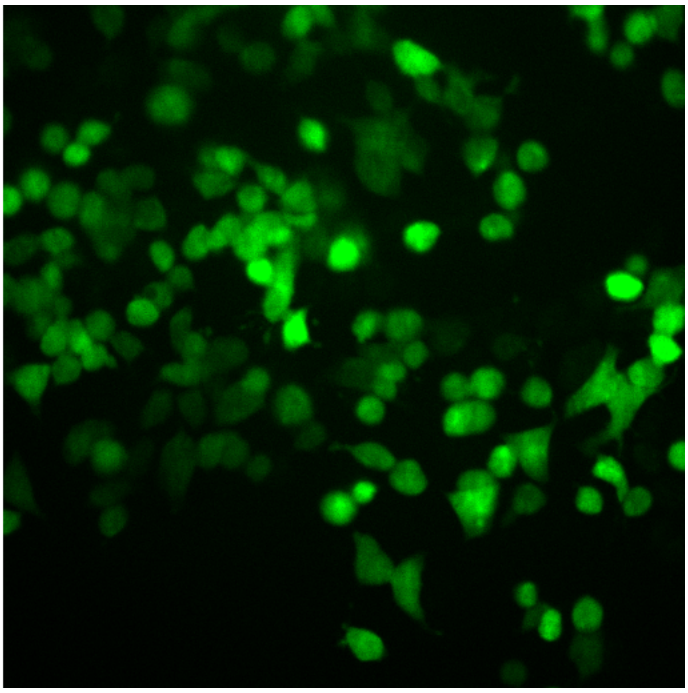

Figure S6: ROS generation in PFOS-treated HepG2 cells (Supplementary for figure 3A)

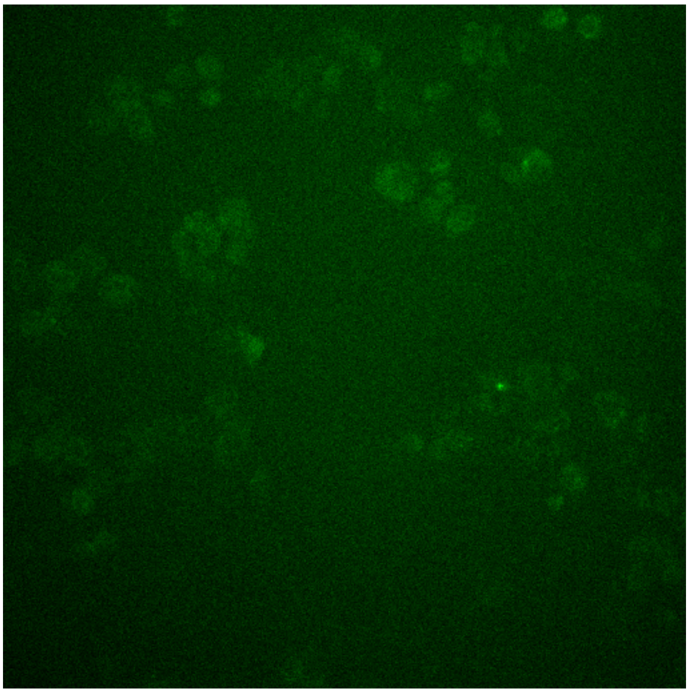

Figure S7: ROS generation in 20% DMSO-treated HepG2 cells (Supplementary for figure 3A)
